# TGFB1 and TGFBR1 polymorphisms and breast cancer risk in the Nurses' Health Study

**DOI:** 10.1186/1471-2407-7-175

**Published:** 2007-09-11

**Authors:** David G Cox, Kathryn Penney, Qun Guo, Susan E Hankinson, David J Hunter

**Affiliations:** 1 Program in Molecular and Genetic Epidemiology, Epidemiology Department, Harvard School of Public Health; 2 Channing Laboratory, Department of Medicine, Brigham and Women's Hospital, Harvard Medical School

## Abstract

**Background:**

Transforming growth factor beta 1 (TGFB1) forms a signaling complex with transforming growth factor beta receptors 1 and 2 and has been described as both a tumor suppressor and tumor promoter. Single nucleotide polymorphisms in *TGFB1 *and a microsatellite in *TGFBR1 *have been investigated for association with risk of breast cancer, with conflicting results.

**Methods:**

We examined polymorphisms in the promoter region of the *TGFB1 *gene as well as the TGFBR1*6A microsatellite in the Nurses' Health Study cohort.

**Results:**

No overall associations between the L10P polymorphism of *TGFB1 *or the *TGFBR1 *microsatellite were detected. However, we observed an inverse association between the -509 C/T polymorphism of *TGFB1 *(p-trend = 0.04), which was stronger and more significant among women with estrogen receptor positive breast cancer.

**Conclusion:**

Polymorphisms in the promoter region of *TGFB1 *are not likely to be associated with large increases in breast cancer risk overall among Caucasian women.

## Background

Transforming growth factor beta 1 (TGFB1) can act as a tumor suppressor by mediating growth arrest via the CDK inhibitors p15^INK4B ^[1] and/or p21^CIP1 ^[2,3] and by inhibiting the expression of c-Myc [4], CDK4 [5,6], and CDC25A [7,8]. Paradoxically, tumor cells have been shown to overexpress TGFB1 [9,10]. This overexpression is thought to induce angiogenesis [11-15] as well as expression of endothelial growth factor, leading to cell proliferation and migration [16,17] and allowing tumor cells to escape from the immune system [18-20].

Polymorphisms (-509 and L10P) in the *TGFB1 *gene have been associated with increased levels of TGFB1 in the serum [21]. The L10P polymorphism has been shown to increase the secretion of TGFB1 *in vitro *[22]. However, studies examining the association between TGFB1 polymorphisms and breast cancer risk have failed to yield a clear picture.

In a prospective cohort of older women, Ziv et al. [23] found a lower risk of breast cancer associated with the C/C genotype as compared to the T/T genotype at the T29C (L10P) SNP (hazard ratio 0.42, 95% CI 0.22 – 0.79). In a population-based case-control study, Dunning et al. [22] found an increase in risk of invasive breast cancer associated with this SNP (Pro/Pro genotype compared to Leu carrier; OR 1.21, 95% CI 1.05 – 1.37). Two other studies found no association between this SNP and breast cancer risk [24,25]. A recent pooled analysis of case-control studies found a moderate increase in breast cancer risk associated with this polymorphism (per variant allele OR 1.08, 95% CI 1.02 – 1.14) [26].

Transforming growth factor beta receptor type I (TGFBR1) is a serine-threonine protein kinase. Though it cannot directly bind TGFB1, it is recruited into a heteromeric complex with the TGFB type II receptor that is able to bind TGFB1 [27]. The signaling complex that results has the two TGFBR1 molecules necessary for the antimitogenic effects of TGFB1 [28]. One common polymorphism in TGFBR1 is a microsatellite in the coding sequence of the gene, corresponding to a variable stretch of alanine residue. *In vitro *studies have shown that TGFBR1*6A (the allele of this microsatellite corresponding to 6 alanine residues) does not respond as well as the normal 9-alanine TGFBR1 to the growth inhibitory signals of TGFB1 [29,30].

The findings of several studies examining the relationship between TGFBR1*6A and cancer have been inconsistent. A meta-analysis of 12 of these studies showed that carrying at least one copy of the 6A allele increased the risk of cancer overall (OR 1.24, 95% CI 1.10 – 1.40) and of breast cancer specifically (1,420 cases and 3,451 controls, OR 1.38, 95% CI 1.14 – 1.67) [31]. We genotyped this microsatellite in the Nurses' Health Study breast cancer nested case-control samples, the largest single study (1,196 cases and 1,677 controls genotyped successfully) analyzed to date, in order to better understand its relationship to breast cancer.

Kaklamani et al. hypothesized that different combinations of the TGFB1 L10P polymorphism and the TGFBR1 microsatellite would produce varying levels of TGFB1 signaling [32]. The authors observed that altered TGFB1 signaling levels altered breast cancer risk (intermediate vs. high signalers OR 1.27, 95% CI 0.93 – 1.74; low vs. high signalers OR 1.69, 95% CI 1.08 – 2.66). We therefore examined this interaction in our study as well.

## Methods

The Nurses' Health Study was established in 1976, when 121,700 female registered nurses between the ages of 30 and 55 completed a self-administered questionnaire on their medical histories and baseline health related exposures. Updated information has been obtained by questionnaires every 2 years. Incident breast cancers were identified by self-report and confirmed by medical record review. Between 1989 and 1990, blood samples were collected from 32,826 of the cohort members. Subsequent follow-up has been greater than 98% for this subcohort. Eligible cases in this study consisted of women with pathologically confirmed incident breast cancer from the subcohort who gave a blood specimen. Cases with a diagnosis anytime after blood collection up to 1 June 2000 with no previously diagnosed cancer except for nonmelanoma skin cancer were included. One or two controls were randomly selected among women who gave a blood sample and were free of diagnosed cancer (excluding nonmelanoma skin cancer) up to and including the questionnaire cycle in which the case was diagnosed. Controls were matched to cases on year of birth, menopausal status, postmenopausal hormone use at blood collection, month of blood return, time of day of blood collection, and fasting status at blood draw. The nested case-control study consists of 1,311 incident breast cancer cases and 1,760 matched controls.

The -509 (rs1800469) and L10P (rs1982073) single nucleotide polymorphisms were genotyped using custom-designed 5' endonuclease assays (Taqman, Applied Biosystems, Foster City, CA; primer and probe sequences available upon request). The TGFBR1*6A microsatellite was genotyped using gel electrophoresis of PCR products. Quality control (QC) replicate samples were included on study plates, with laboratory personnel blinded to both QC and case or control status of the samples. All statistical analyses were carried out using SAS V9.1 (SAS Institute, Cary, NC), with the exception of polytomous regressions [33] and meta-analyses (rmeta package in R). The TGFBR1*6A meta analyses were carried out using prior reports of genotyping results of this polymorphism in breast cancer cases and controls. Power calculations were carried out using Quanto [34]. This study was approved by the IRB of the Brigham and Women's Hospital.

## Results

We did not detect any deviation from Hardy-Weinberg equilibrium at either SNP (p = 0.59 for -509 and 0.49 for L10P in controls). Risk assessments using conditional logistic regression models were similar to unconditional analyses, therefore we will report only the unconditional analyses to increase power. Though no overall association was found between L10P and breast cancer risk, a marginally significant an inverse association between the -509 SNP and breast cancer risk was detected (Table 1). This association was limited to women diagnosed with estrogen receptor (ER) positive tumors (p-heterogeneity in risk between ER+ and ER- breast cancer = 0.002) (Table 2). Compared to controls and using the C/C genotype as a reference, women heterozygous at -509 had an 18% decrease in risk of ER+ breast cancer (OR 0.82, 95% CI 0.67 – 1.00), women homozygous for the T allele had a 38% decrease in risk (OR 0.62, 95% CI 0.42 – 0.90), and there was a highly significant trend in decreased risk across these two genotypes (p = 0.04 for L10P and p = 0.005 for -509). The association was similar among progesterone receptor (PR) positive tumors. No difference in risk was observed upon stratification by menopausal status at diagnosis, body mass index (BMI <30/30+), or postmenopausal hormone (PMH) use (ever/never) for either SNP.

**Table 1:** Association between TGFB1 SNPs and breast cancer risk in the Nurses' Health Study

Genotype	Cases (%)	Controls (%)	OR (95% CI)*
L10L (T/T)	469 (39.6)	613 (37.1)	1.00 (Ref.)
L10P (T/C)	548 (46.2)	797 (48.3)	0.87 (0.74 – 1.04)
P10P (C/C)	168 (14.2)	241 (14.6)	0.92 (0.72 – 1.17)
			p-trend = 0.27

-509 C/C	600 (50.2)	786 (47.3)	1.00 (Ref.)
-509 C/T	506 (42.3)	723 (43.5)	0.89 (0.76 – 1.05)
-509 T/T	89 (7.4)	154 (9.3)	0.76 (0.56 – 1.02)
			p-trend = 0.04

*Unconditional logistic regression controlled for matching factors, family history of breast cancer, age at menopause, age at menarche, BMI at age 18, weight gain since age 18, age at first birth/parity, and personal history of benign breast disease

**Table 2:** Association between the TGFBR1 alanine microsatellite and breast cancer risk in the Nurses' Health Study

Genotype	Cases (%)	Controls (%)	OR* (95% CI)
9A/9A	968 (80.9)	1352 (80.6)	1.00 (Ref.)
6A/9A	207 (17.3)	302 (18.0)	0.95 (0.76 – 1.17)
6A/6A	12 (1.0)	19 (1.1)	0.80 (0.37 – 1.73)
Other	9 (0.8)	4 (0.2)	---

*Unconditional logistic regression controlled for matching factors, family history of breast cancer, age at menopause, age at menarche, BMI at age 18, weight gain since age 18, age at first birth/parity, and personal history of benign breast disease

**Figure 1:**
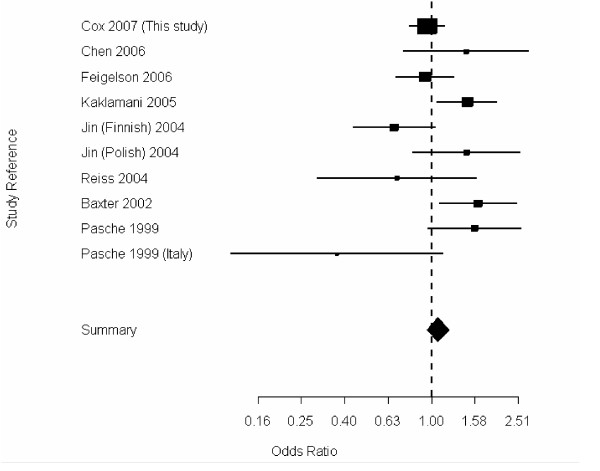
Random-effects meta-analysis of TGFBR1*6A under a dominant model.

The genotype frequencies of the *TGFBR1 *alanine microsatellite (TGFBR1*6A) were similar in cases and controls (Table 2); since there were so few rare variants (<1%), these were removed from the analyses. Again, no deviation from Hardy-Weinberg equilibrium was detected among controls (p = 0.65). Following the classification of high, intermediate, and low signalers proposed by Kaklamani et al., the L10P polymorphism in *TGFB1 *and alleles of the TGFBR1*6A microsatellite were combined. No difference in risk was observed among the high, intermediate, and low signaling types (Table 3), and no difference in risk was observed upon stratifying the cases by estrogen receptor status of their tumors (data not shown). In order to clarify previous reports of association between this polymorphism and breast cancer risk, we have also performed a meta-analysis including our results with previously reported genotyping results of this polymorphism in breast cancer cases and controls. We have separated out all the participating populations (i.e. the two populations represented in Jin et al. [35], genotypes attributed to Reiss in [31], in addition the genotypes attributed to Offitt in [31] were removed, as they were included by Kaklamani et al. [32]) in order to more clearly evaluate and display the data. The summary odds ratio was 1.10 (95% CI 0.89 – 1.38) from the random effects model (Fig. 1), however, there was evidence of significant heterogeneity in risk estimates (p-heterogeneity < 0.01).

**Table 3:** Association between TGFB1 L10P/TGFBR1*6A hypothesized signaling levels and breast cancer risk in the Nurses' Health Study

Signaling level	Cases (%)	Controls (%)	OR* (95% CI)
High	129 (11.7)	185 (11.8)	1.00 (Ref.)
Intermediate	797 (72.1)	1113 (71.3)	0.97 (0.75 – 1.25)
Low	179 (16.2)	264 (16.9)	0.92 (0.68 – 1.26)

*Unconditional logistic regression controlled for matching factors, family history of breast cancer, age at menopause, age at menarche, BMI at age 18, weight gain since age 18, age at first birth/parity, and personal history of benign breast disease

## Discussion

Although the TGFBR1*6A polymorphism has been associated with breast cancer risk in a meta-analysis (with a total of 1,420 cases and 3,451 controls), we did not see any evidence of a relationship between this microsatellite and breast cancer in our large nested case-control study. Our study has 80% power to detect a log-additive per allele odds ratio of 1.27 at the alpha = 0.05 level. We have added our results, and to prior genotyping reports [31,32,35-39]. This new meta-analysis consists of 3,459 breast cancer cases and 4,557 controls. While the summary odds ratio does not show a statistically significant change in risk associated with this polymorphism, there is statistically significant heterogeneity in the risk estimates. One possible explanation of this heterogeneity is variation in the specificity of genotyping methods used. However, the most likely explanation for this heterogeneity is random sampling variation, despite the fact that the studies presented are all composed of mostly Caucasian populations.

Prior studies have shown increases, decreases, or no change in breast cancer risk associated with the L10P polymorphism of *TGFB1*. Recently, the Breast Cancer Association Consortium pooled data from case-control studies examining this polymorphism, including 5,587 breast cancer cases and 6,863 controls, and found a very moderate per-allele increase in breast cancer risk associated with this polymorphism (OR 1.08, 95% CI 1.02 – 1.14) [40]. Our study is underpowered to detect such an association, although the association we observed between this SNP and breast cancer risk was in the opposite direction. If we combine our risk estimate for the L10P polymorphism with those of the Breast Cancer Association Consortium (BCAC), significant heterogeneity in risk estimates (p-heterogeneity = 0.03, random effects model) is observed, and the summary odds ratio would be 1.02, 95% CI 0.88 – 1.17). It is unlikely that population differences would explain the heterogeneity between our results and the BCAC, as in a recent genome wide association scan performed on a subset of the NHS breast cancer cases and controls >99% of the subjects did not have genetic contributions from populations other than Caucasian [41]. One possible explanation for this heterogeneity is that the BCAC is largely composed of prevalent cases, as compared to only incident cases in the NHS, and therefore case-specific variables which may effect the association between this polymorphism and breast cancer risk overall could have different distributions in the BCAC as compared to the NHS. More than likely however, this heterogeneity is due to sampling variation.

More interesting is our observation that TGFB1 polymorphisms are inversely associated with ER+ breast cancers. TGFB1 colocalizes with ERα in mouse mammary epithelial cells, and there is a higher proportion of ERα-positive proliferating mammary epithelial cells in mice with only one copy of the *tgfB *gene, which therefore have significantly lower TGFB1 protein levels [42,43]. This is evidence that TGFB1 may prevent proliferation in ER-positive breast epithelial cells. The T allele of the -509 SNP in TGFB1 has been associated with higher levels of secreted TGFB1 [21]. Our association between this allele and decreased risk of estrogen receptor positive tumors is compatible with the hypothesis that increased TGFB1 levels decrease the potential for proliferation in ER+ breast cells. As these results are not our original hypotheses, our observation that TGFB1 polymorphisms are inversely associated with ER+ breast cancers should be considered hypothesis generating, and needs further replication.

## Conclusion

In conclusion, polymorphisms in the promoter region of *TGFB1 *are not likely to be associated with large increases in breast cancer risk overall among Caucasian women. However, alleles in this region associated with increased TGFB1 levels may reduce the risk of estrogen receptorpositive breast tumors.

## List of Abbreviations

**TGFB1**, Transforming Growth Factor Beta type 1; **TGFBR1**, Transforming Growth Factor Beta type 1 receptor; **OR**, Odds Ratio; **CI**, Confidence Interval; **QC**, Quality Control; **SNP**, Single Nucleotide Polymorphism; **ER**, Estrogen Receptor; **BMI**, Body Mass Index; **PMH**, Postmenopausal Hormone

## Competing interests

The author(s) declare that they have no competing interests.

## Authors' contributions

DGC and KP performed analyses and prepared the manuscript. QG performed statistical analyses and programming. SEH and DJH were responsible for data collection as well as manuscript editing. All authors have read and approve the final manuscript.

**Table 4:** Association between TGFB1 SNPs and breast cancer risk in ER+ breast cancer analyses in the Nurses' Health Study

Genotype	Cases (%)	Controls (%)	OR (95% CI)*
L10L (T/T)	277 (41.3)	613 (37.1)	1.00 (Ref.)
L10P (T/C)	305 (45.5)	797 (48.3)	0.81 (0.66 – 0.99)
P10P (C/C)	89 (13.3)	241 (14.6)	0.79 (0.59 – 1.06)
			p-trend = 0.04

-509 C/C	354 (52.4)	786 (47.3)	1.00 (Ref.)
-509 C/T	276 (40.8)	723 (43.5)	0.82 (0.67 – 1.00)
-509 T/T	46 (6.8)	154 (9.3)	0.63 (0.43 – 0.92)
			p-trend = 0.005

*Unconditional logistic regression controlled for matching factors, family history of breast cancer, age at menopause, age at menarche, BMI at age 18, weight gain since age 18, age at first birth/parity, and personal history of benign breast disease

## Pre-publication history

The pre-publication history for this paper can be accessed here:


